# Origins of Myc Proteins – Using Intrinsic Protein Disorder to Trace Distant Relatives

**DOI:** 10.1371/journal.pone.0075057

**Published:** 2013-09-24

**Authors:** Amir Mahani, Johan Henriksson, Anthony P. H. Wright

**Affiliations:** Department of Laboratory Medicine and Center for Biosciences, Karolinska Institute, Huddinge, Sweden; Universita’ di Padova, Italy

## Abstract

Mammalian Myc proteins are important determinants of cell proliferation as well as the undifferentiated state of stem cells and their activity is frequently deregulated in cancer. Based mainly on conservation in the C-terminal DNA-binding and dimerization domain, Myc-like proteins have been reported in many simpler organisms within and outside the Metazoa but they have not been found in fungi or plants. Several important signature motifs defining mammalian Myc proteins are found in the N-terminal domain but the extent to which these are found in the Myc-like proteins from simpler organisms is not well established. The extent of N-terminal signature sequence conservation would give important insights about the evolution of Myc proteins and their current function in mammalian physiology and disease. In a systematic study of Myc-like proteins we show that N-terminal signature motifs are not readily detectable in individual Myc-like proteins from invertebrates but that weak similarities to Myc boxes 1 and 2 can be found in the N-termini of the simplest Metazoa as well as the unicellular choanoflagellate, *Monosiga brevicollis*, using multiple protein alignments. Phylogenetic support for the connections of these proteins to established Myc proteins is however poor. We show that the pattern of predicted protein disorder along the length of Myc proteins can be used as a complementary approach to making dendrograms of Myc proteins that aids the classification of Myc proteins. This suggests that the pattern of disorder within Myc proteins is more conserved through evolution than their amino acid sequence. In the disorder-based dendrograms the Myc-like proteins from simpler organisms, including *M. brevicollis*, are connected to established Myc proteins with a higher degree of certainty. Our results suggest that protein disorder based dendrograms may be of general significance for studying distant relationships between proteins, such as transcription factors, that have high levels of intrinsic disorder.

## Introduction

Myc family proteins (c-Myc, MycN and MycL in humans) are transcription factors that regulate a large number of genes [1], [2]. Inappropriate activation of Myc proteins is often observed in cancer and it has been suggested that Myc activation is a prerequisite for cancer development [3]. While deregulated expression of c-Myc is involved in the development of many cancers, MycN overexpression is associated primarily with the development of neuroblastoma and some other childhood cancers [4]. MycL is often deregulated in small lung cell carcinoma [4]. Pharmaceutical inactivation of Myc proteins in mice has shown that Myc inhibition may be a promising general approach to cancer therapy [5]. c-Myc and MycN but not MycL are essential for embryonal development in mice [6]. In humans and at least some other primates there is a second MycL protein (MycL2) that is expressed from a processed gene, primarily in testis in humans [7], [8]. Different Myc proteins bind to the same sites in DNA and functional differences between the proteins has been primarily attributed to their differential expression in different cells. This is in part due to the observation that c-Myc and MycN play partly redundant roles during mouse embryogenesis [4] but comparative structure function studies that might reveal differences between the activities of Myc proteins have not been performed systematically. c-Myc promotes cell growth and proliferation and therefore often counteracts cellular differentiation programs. Indeed c-Myc is one of a small number of transcription factors whose overexpression can lead to the re-programming of somatic cells to pluripotent stem cells [9]. Inappropriate activation of Myc in otherwise normal cells leads to apoptosis [10]. MycN and MycL expression is not well correlated with cell proliferation status and these Mycs are thus thought to play an important role in maintaining an undifferentiated state in stem cells and other poorly differentiated cells [4].

Myc binds E-boxes in the DNA of target genes as a heterodimer with the Max protein [11]. Myc has been shown to be a direct regulator of about 15% of protein coding genes, transcribed by RNA Polymerase II, as well as rRNA and tRNA genes, which are transcribed by RNA Polymerases I and III [1], [2], [12], [13], [14]. Activation of the latter is critical for the ribosome biogenesis and increased protein synthesis that drives cell growth. Interestingly, ribosomal protein genes are a major class of Myc-targeted protein coding genes, suggesting that Myc coordinates production of components required for ribosome biogenesis and protein synthesis [15]. Cell growth requires energy and a second major class of Myc target genes is involved in energy metabolism [16]. Myc also targets genes required for apoptosis and senescence [17]. Myc proteins are found in non-mammalian vertebrate and invertebrate organisms [18], [19] and the key role of Myc in cell growth is perhaps most clearly seen in fruit flies, where dosage of the *dmyc* gene is correlated to cell size and consequently the size of the fly [20]. While increased cell size in Myc over-expressing mammalian cells is sometimes seen [21], this is not generally the case because Myc also induces genes required for cell division [22]. In different organisms we may thus find conserved, general Myc functions as well as functions specific to particular groups of organisms.

Myc proteins can be divided into a relatively conserved C-terminal DNA-binding and dimerization domain and a more divergent N-terminal domain. The N-terminal domain plays a critical role for the transcriptional activation and cellular transformation activities of Myc as well as for the control of Myc levels [2]. The conserved Myc Box 1 in the N-terminus is required to keep Myc at its characteristically low level by targeting proteasome-mediated degradation and is a hot spot for cancer associated mutations that stabilize the Myc protein [23], [24], [25]. The similarity of human c-Myc to other human Myc proteins lies between 25–40% at the level of sequence identity. The tertiary structure of the Myc and Max C-terminal domains bound to DNA has been reported [26] as well as formation of alpha-helical conformation in the N-terminal region upon interaction with the TATA-binding protein, TBP [27]. As is typical for transcription factors [28], [29], Myc appears to have a generally disordered protein conformation in the absence of interaction partners. This is important because intrinsically disordered regions within proteins have recently been shown to be enhanced in relatively rare amino acid substitutions associated with Darwinian adaptation [30]. The intrinsically disordered regions within the Myc protein may therefore be significant for the functional adaption of Myc during the evolution of Myc proteins between organisms as well as the Myc family proteins within organisms and during cancer development.

Myc proteins are most clearly characterized by six blocks of conserved residues, which are spread throughout the protein (PRINTS accession number PR00044). These motifs are generally found in vertebrate Myc proteins but in other organisms matches to one or more of the motifs is often insufficient for reliable detection. For example, none of the motifs have been detected in the dMyc protein from fruit flies, even though dMyc has been shown to be a valid Myc homologue experimentally [18]. Sequencing of genomes within the Metazoa and beyond has expanded the realm of organisms known to contain Myc. Myc homologues have been found in many but not all branches of the Metazoa but not in plants or fungi. Intrestingly, a Myc homologue was recently reported in the unicellular choanoflagellate, *Monosiga brevicollis*, which lies phylogenetically close to Metazoans [31]. In this case the Myc motifs defining the C-terminal region are present but it is difficult to identify conserved motifs supporting a common ancestry of the N-terminal domain [32], [33].

Studying Myc proteins from different species can give important perspectives for understanding the role of Myc in normal mammalian cells and cancer cells. However, it is important to determine whether mammalian Myc has been evolved as a complete entity from simple organisms or whether the functionally important N-terminus has been added to the better conserved C-terminal domain at some stage during evolution of the Metazoa. In this work evidence is provided for the evolution of Myc as an intact protein throughout Metazoan evolution and beyond. Further, it is suggested that while Myc protein sequences have diverged greatly, the pattern of intrinsically disordered protein regions is more constrained.

## Results

### Identification of Myc Protein Sequences

**Figure 1:**
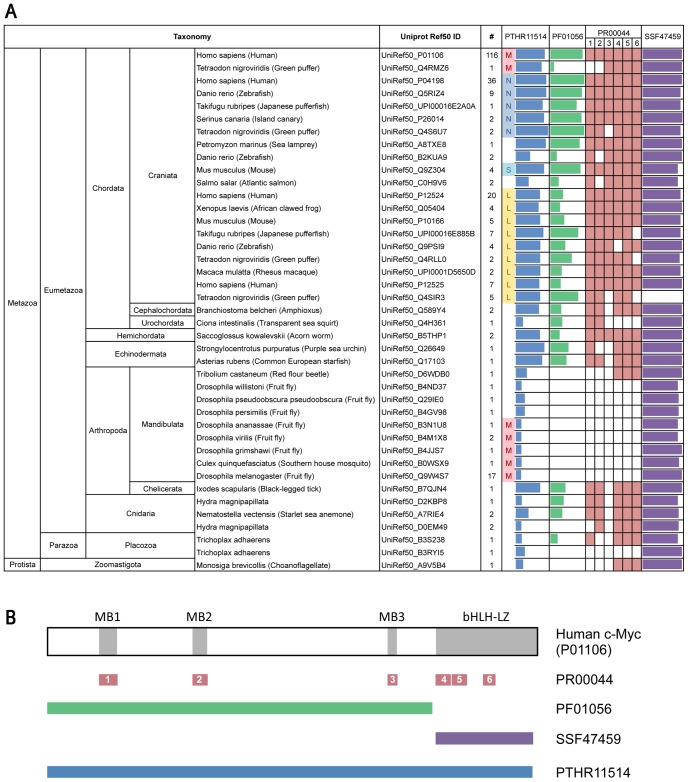
Detection of Myc signature domains in phylogenetically distinct groups of Myc proteins. **A.** Table showing the 41 Uniprot Ref50 groups of Myc proteins selected for the study, including the name of each class, the species from which the sequence representing each class comes and an abbreviated taxonomy to allow location of the classes in a broader classification of organisms. The number of sequences in each class is shown (#). The four columns on the right side show InterPro Scan matches to selected Myc protein signature sequences. The length of the colored bar in the PTHR11514, PF01056 and SSF47459 columns is proportional to the extent of the signature domain match. Some of the PTHR11514 domain matches are sub-chategorised into Myc (M), MycN (N), SMyc (S) and MycL (L) as indicated. **B.** Diagram of the human c-Myc protein showing the positions of conserved Myc boxes (MB1–3) and the basic helix-loop-helix-leucine-zipper (bHLH-LZ) DNA binding and dimerization domain. The positions of each of the selected Myc protein signature sequences are shown below.

The available sequences of Myc proteins from different organisms are highly biased towards sequences from mammals. To allow systematic study of a relatively representative set of Myc proteins from a broader range of organisms BLASTP was used to identify representative groups of Myc proteins in the UniProt Ref50 database. A total of 41 UniRef50 groups were identified as described in the Materials and Methods section, each containing between 1 and 116 Myc protein sequences (Fig. 1A, Table S1). The sequence representing each UniRef50 group was then analysed to identify signature motifs for Myc proteins using the InterProScan algorithm [34]. Fig. 1B shows selected signatures that map to the human c-Myc protein representing the UniRef50_P01106 group. Tabulated data showing identified signatures for all UniRef50 groups is available in Table S2. Fig. 1A shows that all groups, except UniRef50_Q4SIR3, have a good match to the basic helix-loop-helix (bHLH) DNA binding domain motif (SSF47459), which lies within the conserved C-terminal domain of Myc proteins. The extent of the matches to the overall Myc signature (PTHR11514) varies from low, representing homology to the bHLH motif, to high, especially in many vertebrate Myc proteins. The strength of matches to the Myc N-terminus signature (PF01056) is generally high in c-Myc and MycN groups but is lower in MycL groups as well as Myc proteins from invertebrate organisms. In some organisms no match was found to the N-terminal signature. The PR00044 signature is in many ways the most informative signature because it divides up the Myc sequence into six conserved regions that are positioned throughout the Myc sequence (Fig. 1B). While these motifs are almost all present in almost all vertebrate Myc proteins, there is a partial or complete failure to detect them in all of the 19 groups of invertebrate Myc proteins (Fig. 1A). It was concluded that Myc signature motifs are not sufficient for reliable identification of Myc proteins in invertebrate organisms.

### Phylogenetic Analysis of Myc Protein Groups

**Figure 2:**
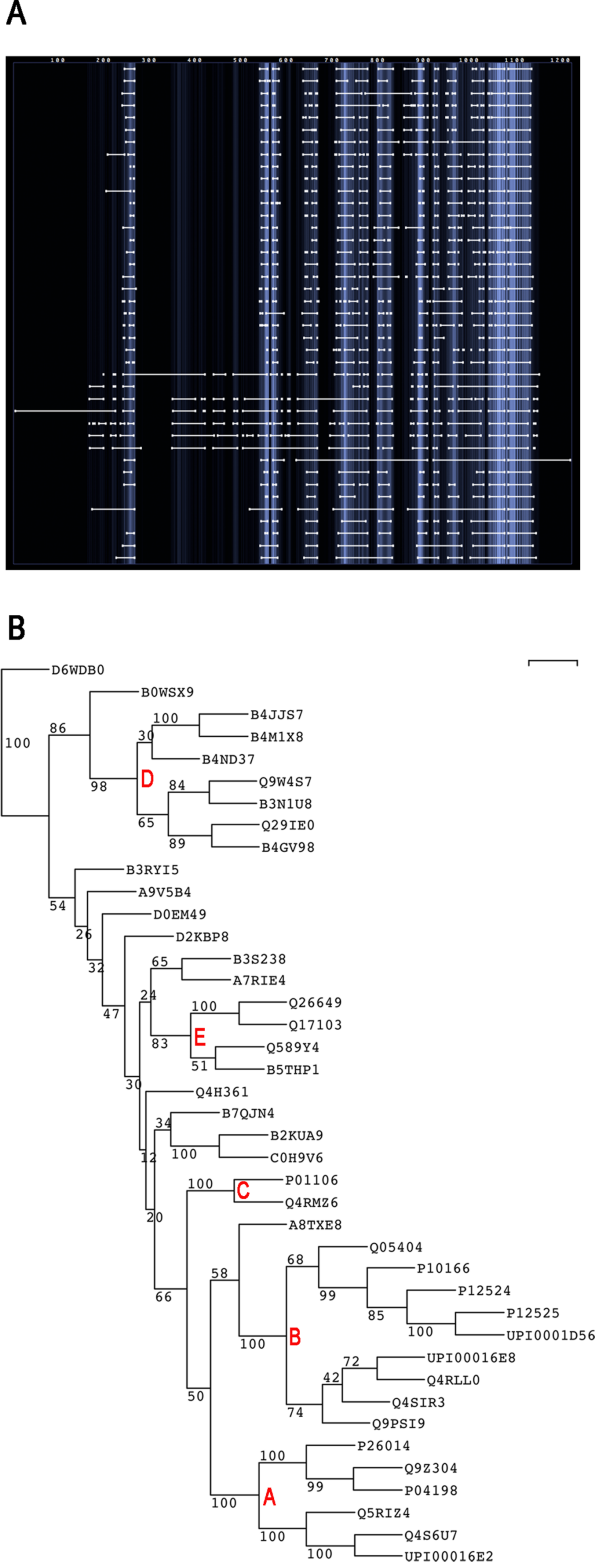
Multiple alignment and phylogenetic analysis of Uniprot Ref50 groups of Myc proteins. **A.** Overview of the ClustalW 2.0 alignment of the representative Myc proteins. The gaps in the N-terminal part of the alignment are primarily due to the longer length of the fruit fly proteins in relation to Myc proteins from other species. **B.** Phylogentic tree showing relationships between the different Myc proteins. Bootstrap values are shown to indicate the level of support for the different parts of the tree topology shown. Well supported clades containing MycN and SMyc proteins (A), MycL proteis (B), c-Myc proteins (C) and dMyc proteins (D), as well as a less well supported clade containing Myc proteins from animals just outside the Craniata (E), are indicated.

To compare the different groups of Myc proteins multiple alignments of the representative protein sequences from each UniProt Ref50 class were made using different algorithms (see Materials and Methods). The best performing algorithm was judged to be ClustalW and the resulting alignment is summarized in Fig. 2A (see Figure S1 for the full multiple alignment). The aligned sequences are characterized by blocks of homology (vertical lines) separated by less well-conserved regions. The extent of less well conserved regions in the N-terminal part of Myc is extended due to sequence expansion within Myc proteins from insects. This accounts for the much longer length of these proteins in relation to Myc from most other species. To systematically investigate the similarity between different Myc proteins the multiple alignment data was used to create a dendrogram (Fig. 1B). The lower part of the dendrogram shows good bootstrap support for clades representing MycN/S (clade A), MycL (clade B) and c-Myc (clade C) proteins. There is also strong support for the clade of fruit fly Myc proteins (clade D) near the top of the dendrogram. The fruit fly sequences are generally much longer than other Myc proteins and were therefore expected to cluster far from the established classes of vertebrate Myc proteins. In between clades A–C and D there is a range of Myc proteins that are much less clearly organized into clades. Many branches close to the root of the tree contain only one protein and bootstrap support for clades containing more than two proteins is generally weak. The only exception is clade E, which contains a number of Myc proteins from organisms close to the division between vertebrates and invertebrates. For the poorly connected proteins the extent and significance of the relationship at the sequence level to the Myc proteins in clades A–D are therefore uncertain, even though several contain easily recognizable Myc motifs in their N-terminus (Fig. 1B) or have been shown experimentally to be Myc homologues [35].

### Level of Sequence Conservation in N-terminal Signature Motifs

**Figure 3:**
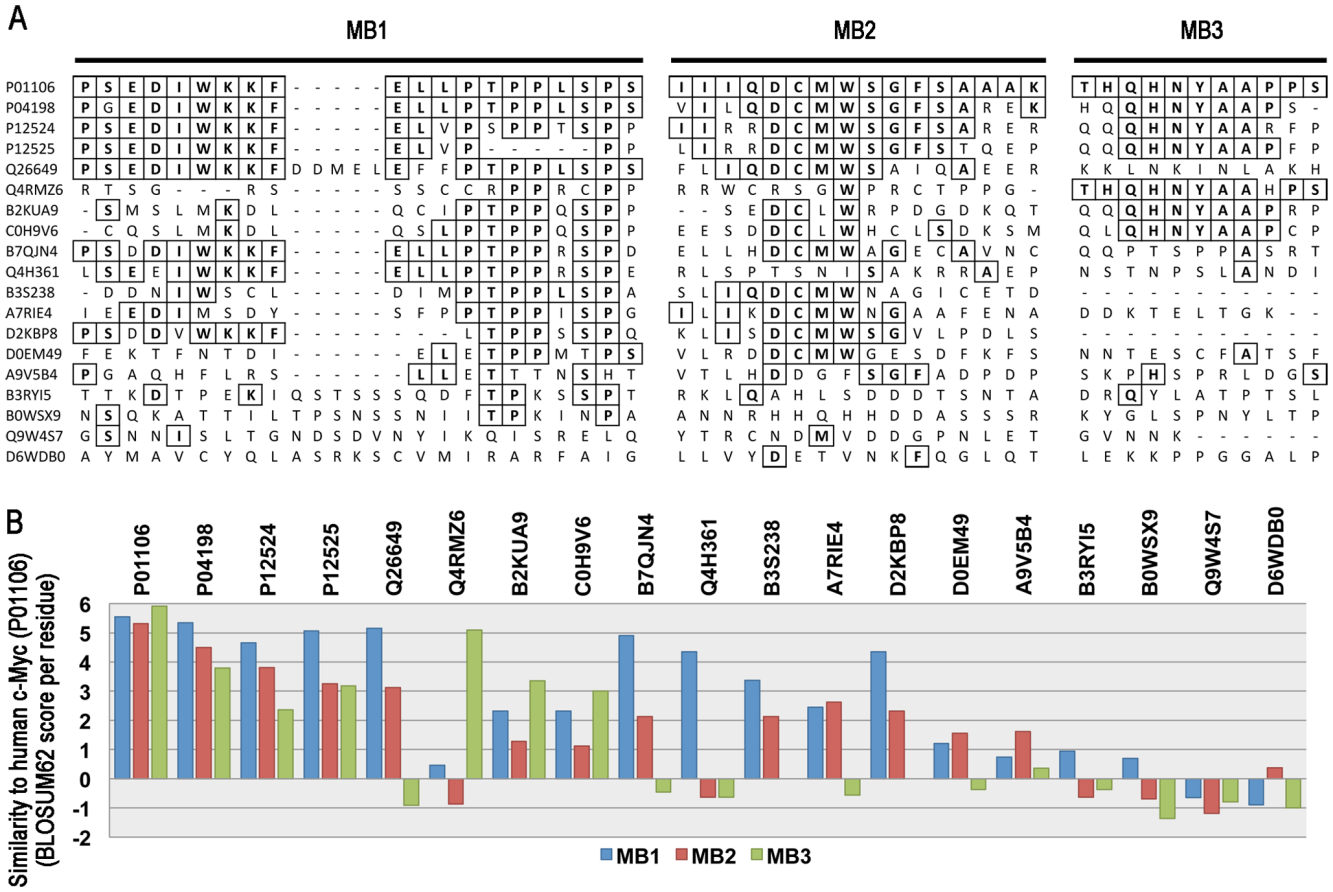
Level of conservation of Myc boxes 1–3 in selected representative Myc proteins that lack an InterPro match to one or more of the regions. **A.** Parts of the ClustalW 2.0 alignment for selected proteins equivalent to Myc boxes 1–3 (MB1–MB3). Human c-Myc (p01106), MycN (P04198) and MycL (P12524 and P12525) sequences are included for reference. Residues enclosed by squares represent residues in the alignment that are identical to human c-Myc. **B.** Bar chart showing the level of conservation of Myc boxes 1–3 for the selected Myc proteins in relation to human c-Myc. The bars show the mean BLOSUM62 score per residue for each residue substitution, in relation to human c-Myc, within Myc boxes 1–3 for each of the selected Myc proteins.

Since the C-terminus of Myc proteins is relatively conserved, the conserved homology boxes (MB1–3, PR00044 1–3 see Fig. 1B) in the Myc N-terminus were studied in more detail in order to further investigate the relationship of poorly connected Myc protein groups to classical Myc protein groups. Fig. 3A shows the appropriate portions of the ClustalW multiple alignment for the poorly connected Myc protein groups in relation to human c-Myc (P01106), MycN (P04198) and MycLs (P12524 and P12525) as well as dMyc (Q9W4S7) from *Drosophila melanogaster*. A number of residues identical to human c-Myc (boxed) can be found in most of the poorly connected sequences, at least for MB1 and MB2. MB3 is much less well conserved. For MB1 the most notable feature of the alignment is the five-residue insertion that is mainly associated with Mandibulata species but which is also seen in B3RY15 and Q26649. The insertion suggests that the MB1 sequences either side of it might have different functions and indeed the C-terminal half of MB1 tends to have a higher level of conservation than the N-terminal part. Interestingly, the C-terminal part of MB1 contains the main recognition sequence for the Fbw7 ubiquitin ligase that plays a critical role in the regulation of c-Myc levels in normal human cells and its dysregulation in some types of cancer [36]. One in four residues in the MB1 and MB2 regions of the *M. brevicollis* protein (A9V5B4) are identical to those in human c-Myc. Although relatively low, this frequency provides evidence to suggest that A9V5B4 and other poorly connected Myc proteins might be related to classically defined Myc proteins throughout their length. A more comprehensive quantitative measure of the relationships of poorly connected Myc proteins to c-Myc was made by calculating the mean similarity of residues in MB1–3 to human c-Myc (P01106) using the BLOSUM62 amino acid similarity matrix. Fig. 3B shows a progressive loss of similarity as divergence from human c-Myc increases but that Myc from the most divergent species (A9V5B4) scores higher for all regions than the fruit fly sequence (Q9W4S7) and some other sequences, thus giving further support to the view that Myc proteins are conserved over the whole length of the protein.

### Comparison of Myc Proteins using Predictions of Structural Disorder

**Figure 4:**
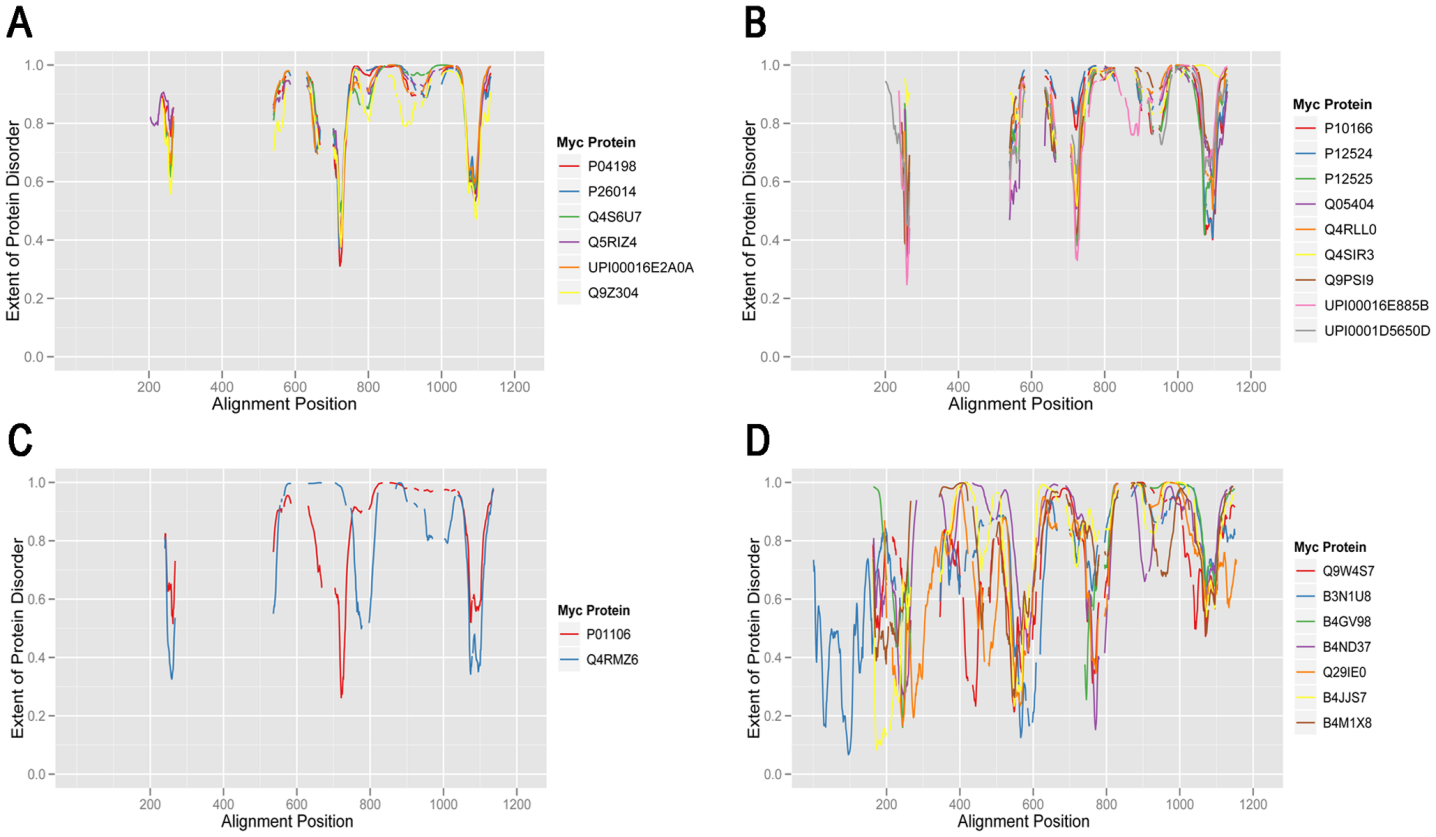
Predicted protein disorder profiles for different clades of representative Myc proteins. A–D. Predicted protein disorder profiles are plotted for proteins in clades identified in Figure 2: MycN/SMyc (A), MycL (B), c-Myc (C), and dMyc (D). Residue-by residue disorder values are plotted against the alignment position of corresponding residues in the ClustalW 2.0 multiple alignment.

Since functionally similar protein structures can in principle be constructed by different amino acid sequences it was reasoned that a useful alternative approach would be to compare Myc proteins at the structural level. Experimental [26], [27], [37] and bioinformatics [29] studies have shown that the c-Myc protein has a low propensity for the formation of structured protein conformation and that, as for many transcription factors, it is one of an increasing number of proteins that are characterized by intrinsic disorder [28]. Disorder prediction scores were calculated using the VSL2P algorithm for the residues of the 41 representative Myc proteins (see Materials and Methods) and mapped on to the ClustalW alignment of Myc proteins (Table S3). While VSL2P was primarily designed as a binary predictor of intrinsic disorder, recent work has shown that the calculated values correlate well with the measured backbone dynamics of tested proteins [38]. Fig. 4 shows disorder prediction scores plotted against alignment position for the different clades of classical Myc proteins identified in Fig. 2B. Clade A, containing mainly MycN sequences, shows a highly conserved pattern of intrinsic disorder prediction. The most variant member of the group is a MycS protein (Q9Z304). Clear similarities are seen between the disorder predictions for the other clades, even though there is more variation than for clade A. Interestingly, the clade C protein, Q4RMZ6, differs significantly from the human c-Myc protein (P01106) with which it is grouped phylogenetically, especially in the N-terminal part. In this case the disorder predictions seem to correspond well with the observation that Q4RMZ6 essentially lacks homology to the MB1 and MB2 regions of c-Myc (Fig. 1A, Fig. 3).

**Figure 5:**
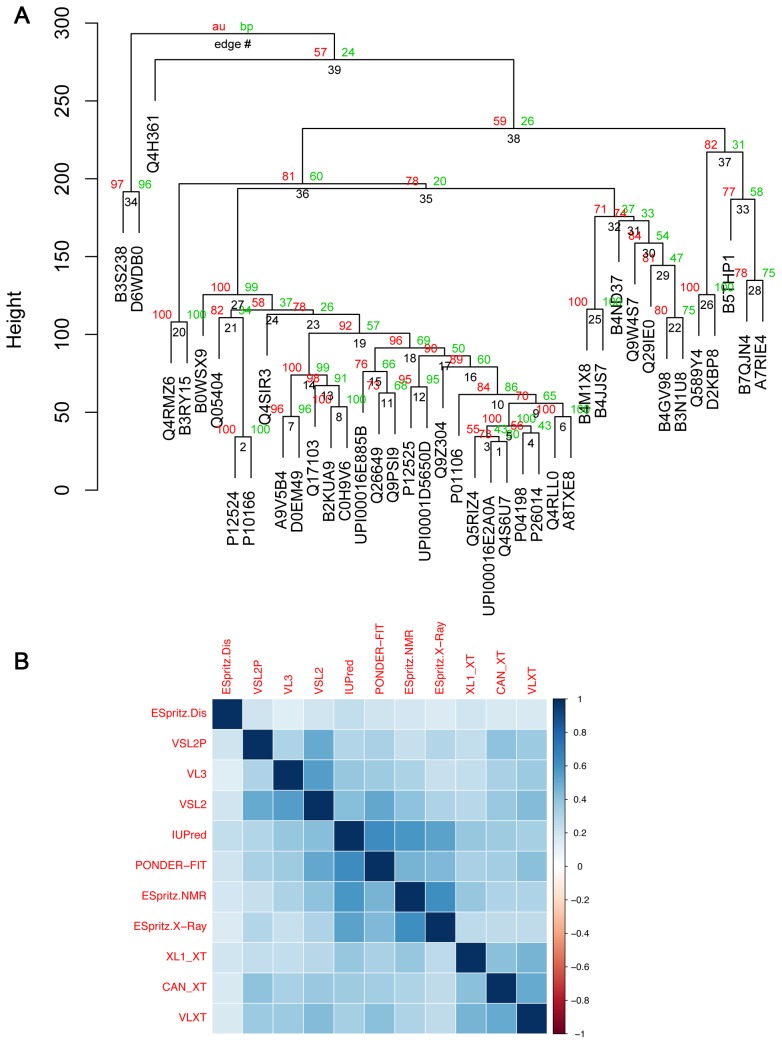
Predicted protein disorder profiles as an approach to identifying relationships between Myc proteins. (A) Dendrogram based on intrinsic disorder values calculated by VSL2P. Approximately unbiased bootstrap values (au) and conventional bootstrap values (bp) are shown as percent values in red and green, respectively. Node numbers (edge #) are shown in black and can be used to identify tabulated bootstrap values (au and bp) and associated standard error values in Table S4. (B) Comparison of dendrograms made using different intrinsic disorder prediction methods. Heat map showing the level of correlation between dendrograms made with different disorder prediction algorithms.

The next step was to systematically test whether disorder predictions can be used to predict relationships between well established groups of Myc proteins. Distances between the aligned predictions for different Myc proteins were used to make bootstrapped dendrograms of relationships between the 41 representative Myc proteins (see Materials and Methods). Fig. 5A shows that intrinsic disorder predictions can be used to recapitulate known phylogenetic groups of Myc proteins (see Table S4 for tabulated bootstrap values and associated standard error values). The clearest example is the grouping of MycN and fruit fly Myc proteins in individual clades. The MycL proteins are contained in a large clade that also contains the MycN clade. There is good statistical support for the robustness of each of these clades. Interestingly, the human c-Myc protein (P01106) is located just outside the MycN clade as is the MycS sequence, which was grouped with MycN in the sequence-based tree. Further, the Q4RMZ6 protein that was clustered together with c-Myc in the sequence-based tree is placed neighboring the fruit fly clade together with one of the *Trichoplax* proteins (B3RY15). VSL2P is one of several different algorithms for predicting intrinsic protein disorder and thus it was relevant to compare the results in Fig. 5A with results from other algorithms. Fig. 5B shows that there is a positive correlation between the results obtained with different algorithms but that some algorithms produce more similar dendrograms than others. The reasons for differences between IDR prediction algorithms in this respect requires further study.

### New Connections between Myc Proteins Based on Protein Disorder Predictions

**Figure 6:**
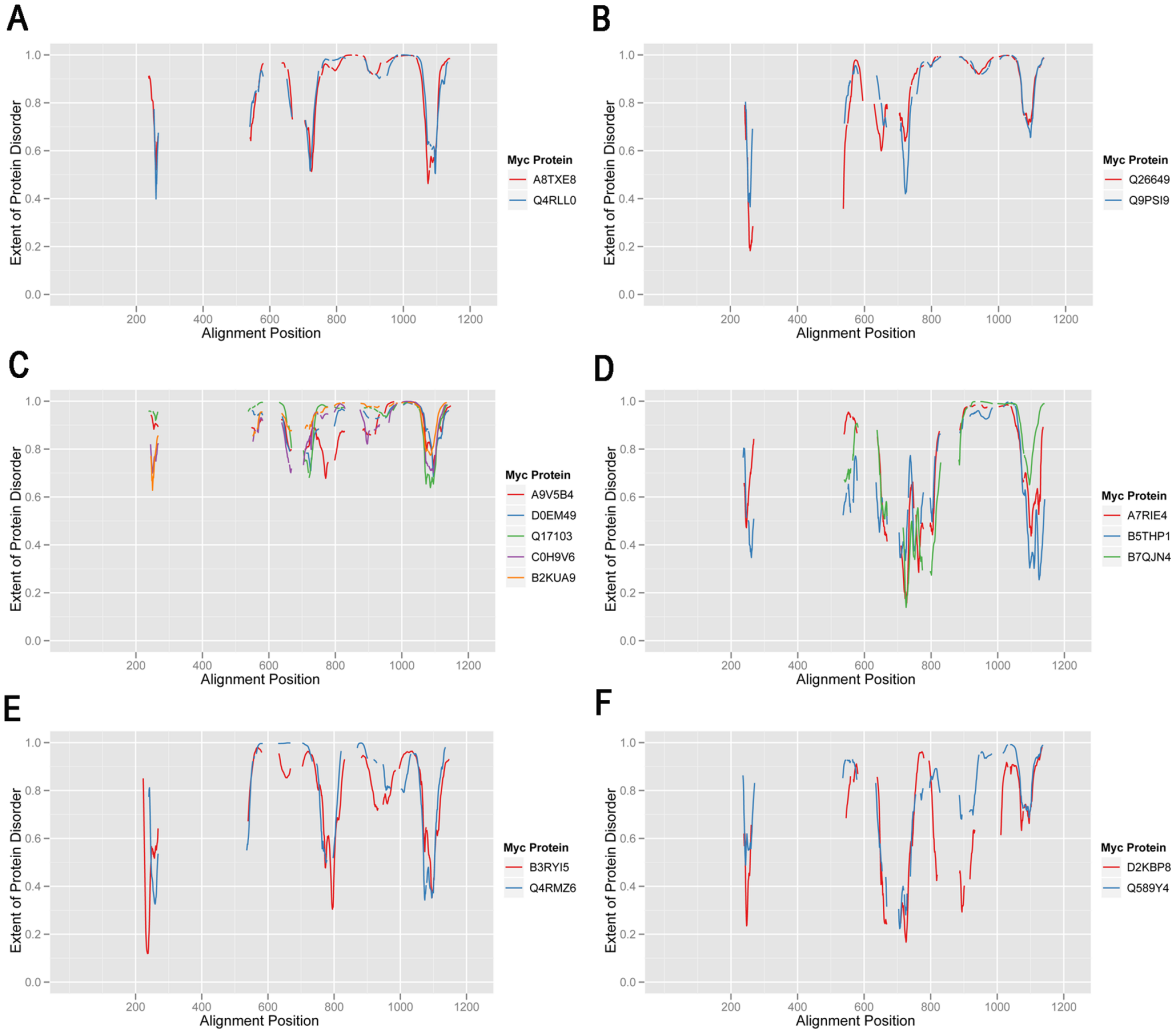
Predicted protein disorder profiles comparing Myc proteins that cluster together in the predicted disorder based dendrogram. A–F. Predicted disorder profiles for different pairs or larger groups of Myc proteins that cluster together in the dendrogram in Figure 5. Annotations are as for Figure 4.

It is interesting to note that Myc proteins that were poorly connected to the sequence-based tree are often much better integrated into the disorder-based tree and that this integration is well supported statistically. These Myc proteins are integrated into the dendrogram in two main ways. First, several are incorporated into the large clade that contains c-Myc, MycN and MycL proteins in the disorder-based tree. These include the sea lamprey Myc (A8TXE8), which is just outside the MycL clade in the sequence-based tree. Fig. 6A shows the similarity of the disorder prediction for A8TXE8 in relation to its nearest neighbor in the disorder-based tree, the Q4RLL0 MycL protein. Similarly, the sea urchin protein (Q26649) clusters together with the Q9PSI9 MycL protein (Fig. 5), which has a very similar disorder prediction pattern (Fig. 6B). In addition to these single protein additions the c-Myc, MycN, MycL clade also contains a small clade of five additional proteins (Fig. 5). This clade is composed of two fish proteins (B2KUA9, C0H9V6) that are not classified with regard to what type of Myc they represent (c, N or L) as well as three proteins from starfish (Q17103), *Hydra* (D0EM49) and choanoflagellate (A9V5B4). Choanoflagellates lie outside the Metazoa and are the organisms most distant from mammals in which Myc proteins have been reported. Interestingly, the A9V5B4 protein clusters most closely with D0EM49, which has been shown to have Myc-like functions in *Hydra* [35]. Fig. 6C shows that this clade of proteins shows similar patterns of disorder, where a major characteristic is the relatively high disorder prediction for Myc regions that are predicted to have lower levels of disorder in other Myc proteins.

The second way in which poorly connected Myc proteins are integrated into the disorder-based tree is that they form clusters of two or more proteins that flank the large c-Myc, MycN, MycL, dMyc clade. For example, the sea anemone (A7RIE4) and tick (B7QJN4) proteins are robustly found clustered together with the Acorn worm (B5THP1) protein from the hemi-chordates (Fig. 5A). The similarity in the predicted disorder pattern for the three proteins is shown in Fig. 6D. Other interesting connections include the *Trichophlax* (B3RTI5) and *Hydra* (D2K8P8) proteins that are clustered together with the Green puffer fish (Q4RMZ6) and *Amphioxus* (Q589Y4) vertebrate Myc proteins (Fig. 5, Fig. 6E and F). It can be concluded that predicted intrinsic protein disorder predictions are a useful complement to protein sequences for determining relationships between evolutionary divergent Myc proteins.

### Relationship between Intrinsic Disorder and Eukaryotic Linear Motifs in Myc Proteins

The relative conservation of intrinsic disorder patterns in Myc proteins in relation to their amino acid sequences suggests the existence of conserved disorder-related functional characteristics that are not always coupled to conserved amino acid sequences. We therefore investigated whether there was a significant tendency of eukaryotic linear motifs (ELMs) to be associated with regions with higher propensities for intrinsic disorder throughout the set of 41 representative Myc proteins. ELMs are short peptide sequences that are associated with functions, such as being sites of posttranslational modification and protein interaction, and are available from the ELMs database [39]. The positions of ELMs in Myc proteins (Table S5) showed a significant tendency of ELMs to be localized in protein regions with a higher propensity for intrinsic disorder, predicted by VSL2P (p = 4.0E-5), Espritz-NMR (p = 1.3E-9) or IUPred (p = 2.7E-4). At the level of individual ELMs, several ELMs representing features known to be of importance for Myc function [40] show a significant tendency to be associated with protein regions that have high propensity for intrinsic disorder. These include binding sites for the Fbw7 ubiquitin ligase (1.06E-6) as well as sites post-translationally phosphorylated by Gsk3 and cyclin-dependent protein kinases (<1.00E-99 and 4.03E-7, respectively).

Since many identified ELMs represent interaction sites for Myc binding proteins or proteins that post-translationally modify Myc, we compared the association of ELMs with ANCHOR sequences detected in human Myc proteins. ANCHOR sequences are derived from intrinsic disorder predictions (IUPred) and are thought to correspond to protein interaction sites [41]. For c-Myc (P01106, Table S6), MycN (P04198, Table S7) and MycL1 (P12524, Table S8) the count frequencies for different ELM categories within ANCHOR regions does not vary significantly from their frequency overall (Table 1). However, the ELM category frequencies within ANCHOR regions for MycL2 (P12525, Table S9) differ significantly from the overall frequency. Table 1 shows that the P12525 protein has fewer ANCHOR-related ELMS but that ELMS in the LIG category of ligand-binding sites appear to be preferentially maintained in relation to other ELM categories. Comparison of the two MycL proteins shows that the number and extent of ANCHOR regions is reduced to about half in P12525 compared to P12524, perhaps corresponding to the lower median intrinsic disorder score for this protein (Table 2). Table 2 also shows that the apparent association between the LIG ELM category and ANCHOR sequences in P12525 is significant. The significance of this finding in the relation to the evolution and function of MycL proteins is discussed further in the Discussion.

**Table 1:** Altered relative frequency of ELM category hits associated with ANCHOR regions in the transcript-copied MycL protein (P12525).

	P01106	P04198	P12524	P12525
Total ELM number (ANCHOR associated)	138(55)	110(73)	80(29)	85(13)
- CLV (ANCHOR associated)^1^	20(9)	11(6)	13(4)	13(0)
- LIG (ANCHOR associated)^1^	37(16)	45(35)	33(14)	30(9)
- MOD (ANCHOR associated)^1^	78(29)	51(32)	32(9)	40(3)
- TRG (ANCHOR associated)^1^	3(1)	3(0)	2(2)	2(1)
p–value^2^	0.920	0.340	0.473	0.025

1 CLV = cleavage sites, LIG = ligand-binding sites, MOD = post-translational modification sites, TRG = targeting sites.

2 CHI squared test, 2 tailed, df = 3.

**Table 2:** Selective association of the ligand-binding ELM category with ANCHOR regions in the transcript-copied MycL protein (P12525).

	P12524	P12525
Length (amino acid residues)	364	357
Amino acid identity (%)	69.5	
Median VSL2P score	0.92	0.86
Total ANCHOR regions	7	3
Length ANCHOR regions (%)	132 (36)	69 (19)
Total ELM number (ANCHOR associated)	80 (29)	85 (14)
p–value, CLV enrichment in ANCHORs^1^	0.76	0.20
p–value, LIG enrichment in ANCHORs^1^	0.36	0.01
p–value, MOD enrichment in ANCHORs^1^	0.24	0.08
p–value, TRG enrichment in ANCHORs^1^	0.13	0.28

1 Significance of ELM category association with ANCHOR, Fisher exact test (2-tailed).

CLV = cleavage sites, LIG = ligand-binding sites, MOD = post-translational modification sites, TRG = targeting sites.

### The Effect of Cancer-related Amino Acid Substitutions on the Intrinsic Disorder of Myc

**Figure 7:**
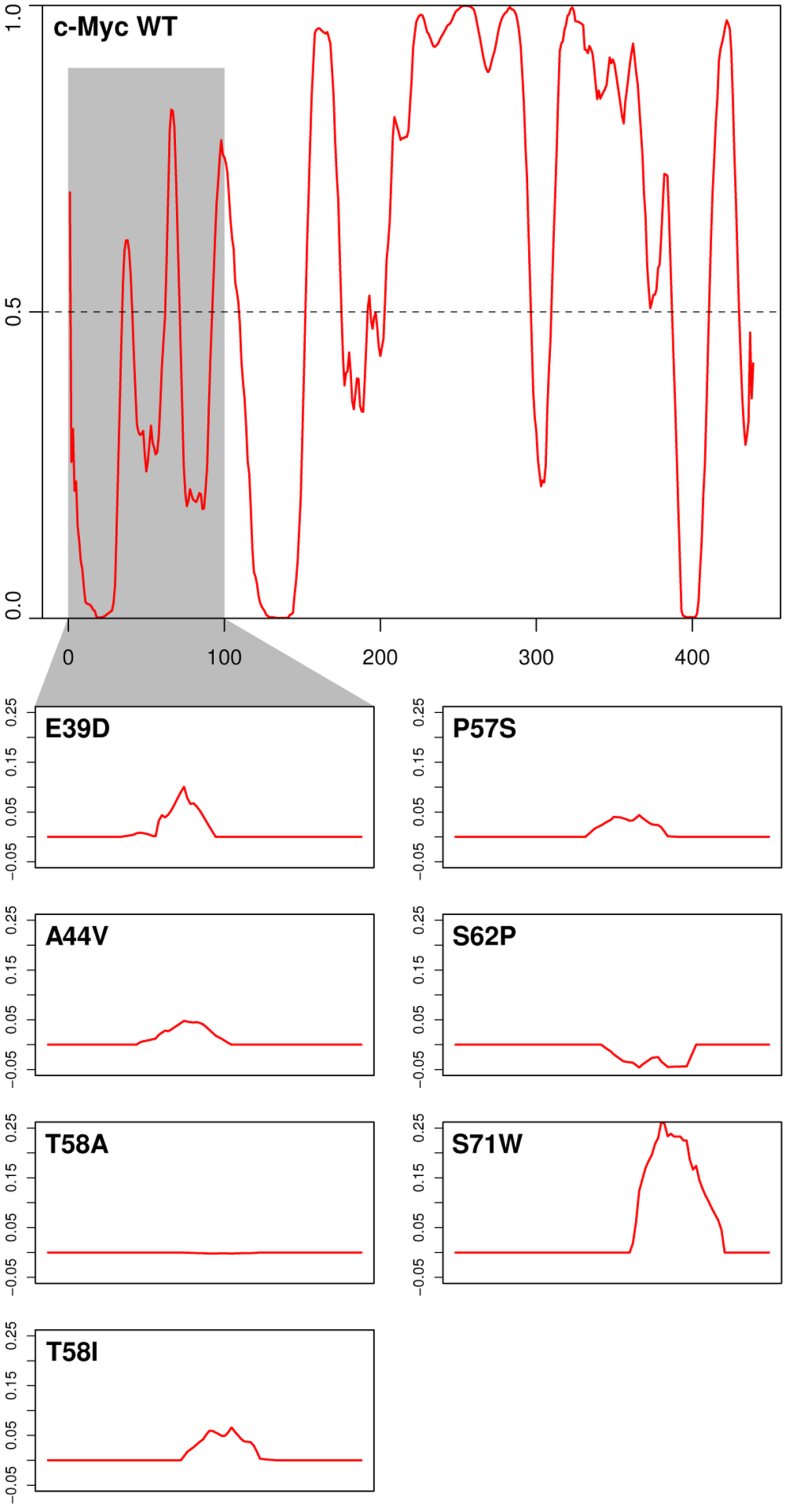
Effect of cancer-related, functionally significant mutations on predicted disorder profiles. The upper panel shows the disorder profile for human c-Myc (P01106) calculated using the VLXT algorithm. The lower panels show difference disorder profiles (mutant values minus wild type values) for the mutant c-Myc proteins described in Table 3. The difference profiles are sown for the shaded region in the upper panel.

There is a hot-spot for cancer-related amino acid substitutions in the N-terminus of c-Myc, which has been shown experimentally to be intrinsically disordered [27], [37], [42]. Several of these mutations have been shown to cause changes in the function of c-Myc in relation to its role as an inducer of cell growth, proliferation, transformation or apoptosis (Table 3) [42]. As a first step to understanding possible ties between intrinsic disorder of Myc proteins and their function, we studied the effect of such functionally relevant substitutions on predictions of intrinsic disorder. Figure 7 shows the disorder profile of c-Myc (P01106) predicted by the VLXT algorithm together with difference plots in which disorder scores for mutant proteins are shown after subtraction of wild type values. Most mutations tend to increase the level of predicted disorder, although there are exceptions (S62P and T58A). Interestingly, T58I has been shown to have a larger functional effect than T58A [42], which correlates with the larger effect of substituting isoleucine compared to alanine at this position.

**Table 3:** Functionally significant amino acid substitutions in human c-Myc that occur in cancer.

Residue	Substitution^1^	Number of Cases^1^
E 39	D	11
A 44	V	6
P 57	S	8
T 58	I	11
T 58	A	2
S 62	P	3
S 71	W	1

1 Described in Chang et al [42].

## Discussion

Understanding the evolutionary origin of proteins as well as adaptive events that influence their function can give important perspectives for understanding their role in human health and disease. However, finding the point of origin of existing proteins can be difficult. In the case of Myc this process is complicated by the fact that Myc proteins appear to have evolved from a larger family of basic helix-loop-helix proteins to which the Myc C-terminus is homologous. The Myc N-terminus is characterized by homology blocks that are critical for Myc function in mammals but there is no clear expectation about whether these sequences should be important in more distantly related Myc proteins. It is at least hypothetically possible that the N-terminal signature sequences where added during metazoan evolution to a Myc progenitor protein that lacked them. Importantly, our BLAST search strategy was designed to detect proteins with homology to Myc outside the conserved basic helix-loop-helix leucine zipper region.

Outside of the classical groups of mammalian Myc proteins (clades A–C in Fig. 2B) species from the Hemichordata and Echinodermata cluster together with the vertebrate Myc from *Amphioxus* (Q589Y4) in clade E (Fig. 2B). These proteins contain most of the N-terminal Myc signature sequences (Fig. 1A). The more distantly related Cnidarian Myc proteins contain most of the Myc signature sequences, though not MB3. These proteins are however, not well clustered in the phylogenetic tree, where they either form single protein clades or are clustered together with proteins in which the N-terminal signature sequences are less well conserved or absent (Fig. 1A). Importantly, one of the Cnidarian sequences from *Hydra* (D0EM49) has been shown to have Myc-like functions in experimental studies [35]. The existence of N-terminal Myc signature sequences in both Cnideria and vertebrates suggests that the common progenitor of these Metazoan branches also had the Myc signature sequences. The absence of signature sequences in branches of the Arthropods therefore suggests divergent evolution of Myc in insects and some other related species. This can explain the almost complete lack of Myc signature sequences in these species (Fig. 1A) as well as their peripheral clustering in clade D of the phylogenetic tree (Fig. 2B). Experimental studies of the fruit fly, *Drosophila melanogaster*, show however that the dMyc protein (Q9W4S7) does have Myc-like functional properties and thus Myc proteins are present in insects where they play important roles in processes such as cell growth regulation, just as they do in vertebrates. It should be noted that Myc proteins do not seem to be present in all branches of the Metazoa. At least in some nematodes the Myc protein seems to have been lost since it has not been discovered either at the level of sequence analysis or genetic studies in well-studied species such as *Chaenorhabditis elegans*. A complete picture of the evolution and loss of Myc proteins will require studies of a broader range of organisms within and beyond the Metazoa.

It is of interest to determine whether Myc proteins pre-date the development of multi-cellular animals and it therefore important to determine the status of Myc-like proteins from species in the Parazoa and Zoomastigota which span the branch point of Meazoans from other life forms. These proteins include the *Trichoplax adhaerens* proteins (B3S238 and B3RYI5) and the non-Metazoan protein (A9V5B4) from *Monosiga brevicollis* [31], [33], [43]. These proteins almost completely lack matches to the Myc N-terminal signature motifs (Fig. 1A) and are poorly connected to the phylogenetic tree (Fig. 2B). Detailed study of the multiple alignment in the MB1–3 signature regions shows that all three proteins have few if any residues that match MB3. This is also seen for proteins from species that are much more closely related to vertebrates. In the MB1 and MB2 regions all three proteins have several residues identical to those in vertebrates. In MB1 the identical residues are more prevalent in the C-terminal half of the motif, which is the recognition site for the Fwb7 ubiquitin ligase that negatively regulates the level of mammalian Myc proteins. Indeed the key threonine and serine residues (equivalent to T58 and S62 in human c-Myc), whose phosphorylation regulates Myc degradation, are conserved in all three proteins.

The library of possible protein sequences is redundant in relation to the range of possible protein structures that can be formed. Studies of protein homology can thus often be aided by comparing the structures of proteins. For transcription factors like Myc this approach is difficult because transcription factors have a low propensity for structure formation. This characteristic appears to be important for their ability to interact with and recruit a large range of structurally unrelated partner proteins during gene regulation [44], [45]. Computer algorithms can be used to reliably predict the protein disorder propensity along the length of proteins. By analogy to the situation in structured proteins, it could be reasoned that disorder patterns might be conserved in Myc proteins even if the sequence is not and there is some evidence that this is sometimes the case for other proteins [46], [47]. Conservation of intrinsic disorder patterns might thus be useful for identifying distant relatives of vertebrate Myc proteins. This appears to be the case for Myc proteins and as shown in Figs. 5 and 6, several of the single clade proteins from sequence-based tree could be clustered together with more Myc-like proteins. This is true for the non-Metazoan *Monosiga* protein (A9V5B4) that clusters together with the experimentally verified Myc protein from *Hydra* (DOEM49) in a larger clade containing starfish and fish proteins. This clade is characterized by relatively high levels of disorder throughout the proteins in comparison with other Myc proteins. Other poorly connected single clade proteins from the sequence-based tree that are better integrated into the disorder-based tree are the B3RYI5 and D2KBP8 proteins from *Trichoplax* and *Hydra*, respectively. In two cases (A8TXE8 and Q26649) proteins from outside the classical Myc families can be closely coupled to particular MycL proteins on the basis of the predicted disorder patterns. Comparison of dendograms produced using disorder data from different algorithms showed that all dendrograms were positively correlated but that there were also clear differences, primarily between different groups of algorithms. Overall, the classification of the classical vertebrate Myc families and of the fruit fly Mycs tends to be more robust across algorithms while the grouping of the invertebrate Mycs is more variable. While no direct comparison has been made with the variation associated with different approaches to sequence-based dendrograms, protein disorder predictions should not be regarded as a replacement for sequenced-based methods but they provide a useful complementary approach for measuring relationships between Myc proteins. While further studies are needed to fully understand parameters that influence the use of intrinsic disorder data for identifying relationships between proteins, it is likely that this approach can be generalized for studies of at least some other transcription factors and non-transcription factor protein classes that lack a strongly defined secondary structure.

MycN and MycL protein groups each have both common and distinct intrinsic disorder characteristics (Fig. 4 AB). Even the fruit fly Myc proteins (Fig. 4D) that are divergent in sequence compared to classical Myc proteins, have protein disorder similarities to MycL proteins (Fig. 4B). In both cases there are regions of reduced disorder in similar regions of the alignment (eg. 550–600, 700–800, 1050–1100) and a variably reduced region of disorder at around 850–950). It has been suggested that regions of reduced disorder in generally disordered proteins are important for interaction with partner proteins or DNA [41], [48]. These regions often contain ELMs that are shared, more or less, between the groups of MycN and MycL proteins but in some cases ELMs are almost specific for MycN or MycL, indicating possibly important functional differences between the proteins. An example is the ELM, MOD_GSK_1, which occurs in all 5 MycN proteins in the alignment interval 700–800 (n = 17, median number per protein = 4, range 1–5). The same ELM occurs in the equivalent region of the 9 MycL proteins in only two proteins (single occurrence in each case). With regard to binding of partner proteins, comparison of the two human MycL proteins is of interest. As explained in the Introduction the gene encoding MycL2 lacks introns and is thought to be evolved from a progenitor of MycL1 by reverse transcription of MycL1 mRNA. MycL2 is expressed in a much more limited range of tissues than MycL1, primarily testis, which might be a reflection of functional specialization of MycL2. This could account for the lower content of ANCHOR regions and ANCHOR associated ELM sequences in MycL2 compared to MycL1. In the context of this reduction in ANCHOR content it is of interest that the LIG ELM category has been preferentially maintained in the MycL2 ANCHOR regions in relation to the other ELM categories. This is consistent with a role of MycL2 ANCHOR regions in the binding of interaction partners.

Given the link between intrinsic disorder and protein interaction, it is thus possible that protein disorder profiles represent a basic architecture for unstructured proteins that tends to be evolutionarily constrained to a higher degree than the sequence itself, as has been suggested previously [46], [47]. It is, for example, possible that adaptive mutations could occur without infringing this general disorder pattern constraint in order to allow protein interaction with different repertoires of partners. Indeed, evidence has been shown previously to indicate that adaptive mutations occur more commonly in disordered regions of proteins rather than regions of defined secondary or tertiary structure [30] and that disordered regions evolve more quickly [49], [50]. Alternatively, adaptive mutations might change the disorder properties of disordered proteins in order to change their propensity for interaction with partners and thus their functionality. Of course, both possibilities may occur in different situations and in the context of different evolutionary time-scales. In this context it is interesting to note that functionally relevant cancer mutations in c-Myc generally cause changes in the local intrinsic disorder of c-Myc.

## Materials and Methods

### Identification of Candidate Myc Proteins

Proteins homologous to Myc were identified using BlastP [51] with human c-Myc (P01106) as the query sequence and the UniRef50 database of protein sequences [52]. UniRef50 groups highly similar sequences and chooses a single representative sequence to represent the group. Thus only the representative sequence is returned by BlastP. This helps to compensate for the imbalance in the number of protein sequences available from different groups of organisms. Related groups of non-Myc proteins, usually with homology to the bHLHZ domain that is relatively conserved in several other protein classes [53], were excluded by choosing only BlastP matches with a score greater than 140 and a match length of greater than 300 amino acid residues. Importantly, these thresholds maintained distantly related but experimentally validated Myc homologoes such as dMyc (Q9W4S7).

### Identification of Myc Signature Motifs

Myc-related motifs were detected in candidate Myc proteins using the InterPro Scan algorithm [34] as implemented at http://www.ebi.ac.uk/Tools/pfa/iprscan/. For each of the 41 UniRef50 representative candidate proteins the text output containing details about matches to InterPro motifs, and available as “tool output”, was saved (Table S2).

### Multiple Alignment and Phylogeny of Myc Proteins

Multiple protein alignments were constructed using three different algorithms. ClustalW 2.0 was used as implemented at http://mobyle.pasteur.fr using default settings, including use of the Gonnet series protein weight matrix [54]. The T-Coffee [55] and MUSCLE [56] algorithms were used with default settings as implemented in the BioX bioinformatics software package (http://www.ebioinformatics.org). The BioX package was also used to make overview pictures of multiple alignments. Distance based phylogenetic trees were made from multiple protein alignments using the protdist (bootstrap = 100) and neighbor algorithms as implemented at http://mobyle.pasteur.fr. Inspection of the trees produced by the three alignment methods used showed only minor differences in tree topology (data not shown). The trees were further evaluated by their ability to align residues within conserved Myc boxes 1 and 2 within the otherwise poorly conserved N-termini of Myc proteins. The algorithms were similarly successful but ClustalW 2.0 was preferred since it introduced fewer gaps in the aligned sequences than the other algorithms.

### Comparing the Intrinsic Disorder Properties of Myc Proteins

The level of intrinsic protein disorder was predicted using the VSL2P algorithm [57] with an output window length equal to 1, as implemented at http://www.dabi.temple.edu/disprot/predictor.php. The algorithm is one of the best performing disorder predictors with a prediction accuracy over 80% [58]. Other algorithms used for intrinsic protein disorder were ESpritz.Disprot [59], ESpritz.X-Ray [59], ESpritz.NMR [59], IUPred [60], [61], PONDER-FIT [62], PONDR® VLXT [63], [64], PONDR® CAN_XT [63], PONDR® XL1_XT [62], PONDR® VSL2 [57], PONDR® VL3 [65], ANCHOR [41], [66]. To allow comparison of the predicted disorder profiles of different Myc proteins the residue-by-residue predicted disorder values were substituted into multiple protein alignments in place of the equivalent amino acid residues. To do this the IDR output was matched with a multiple alignment using a custom Java program, taking a FASTA file containing the alignment and a CSV-file containing IDR-scores as input. For visualization and comparison, individual protein entries were extracted from the resulting aligned disorder matrix and disorder predictions were plotted against their alignment position using the R ggplot2 package [67] with line colors chosen from the RColorBrewer package available from http://cran.r-project.org. Predicted disorder based trees were made from the aligned disorder matrix (above) using the R pvclust package [68], with 10,000 bootstrap replications. The pvclust package uses the R hclust hierarchical clustering function and delivers a conventional bootstrap p-value (BP) as well as an approximately unbiased (AU) p-value from multi-scale bootstrap resampling, which provides an improved p-value estimate. P-values are expressed as percent where 100% is regarded as the most reliable support for clustering. The seplot and print functions in pvclust were used to determine the standard error for the p-values. Euclidean distance measures were used. To compare dendrograms made using the output from different protein disorder prediction algorithms, the distance d(A,B) between two genes A and B was defined to be the number of edges along the path in-between. A vector was then created using d(gene_i_,gene_j_) for each dendrogram. The dendrograms could then be compared by Pearson correlation, using R hclust (euclidian distance) for ordering along the axis. The genes themselves were in this case not clustered according to the Euclidian metric, but rather using d′ = 1−m(g_i_,g_j_)ρ(g_i_,g_j_), where m is the fraction of overlap, and ρ is pearson correlation.

### Analysis of Intrinsic Protein Disorder in Relation to Eukaryotic Linear Motifs (ELMs)

ELMs were identified in Myc protein sequences by parsing a downloaded HTML-file from elm.eu.org [39] using a custom Java program to harvest ELM region information into a CSV output file. The positions were simultaneously remapped using the FASTA file containing the ClaustalW 2.0 multiple alignment. The overall relationship between the location of ELMs and intrinsic protein disorder was evaluated by extracting all IDR scores for those positions covered by an ELM (not double-counted when overlapped by multiple ELM). The complementary set of IDR scores outside ELM regions was also formed. A non-parametric Wilcox test was performed to assess if the IDR-score was higher inside the ELM regions.

The incidence of individual ELMs together with intrinsic protein disorder was simplified for computational reasons. Assuming each ELM covers a small fraction of all amino acids, all IDR-scores for overlapping positions were collected. This was compared with the set of all IDR-scores, including those covered by the ELM. This will in all cases give a minor underestimation of any enrichment. The p-values where calculated by Wilcox non-parametric comparison of the mean IDR-score. The R package sqldf was used for all ELM-IDR comparisons [69]. The incidence of ELMs in relation to ANCHOR sequences [41] was determined using the webserver available at http://anchor.enzim.hu [66]. The relative frequency of ELM categories associated with ANCHOR regions was compared to the overall frequency using a Chi squared test. The significance of overlaps between ELM categories and ANCHOR sequences was evaluated using Fisher’s Exact test.

## Supporting Information

Figure S1 **ClustalW multiple alignment of 41 representative Myc proteins.**
(TXT)

Table S1 **Sequences constituting UniRef50 groups of representative proteins.**
(TXT)

Table S2 **Signature motifs identified in representative Myc proteins using InterPro Scan.**
(TXT)

Table S3 **Table showing VSL2P intrinsic disorder scores mapped onto the ClustalW multiple alignment of representative Myc proteins.**
(TXT)

Table S4 **Probability values and associated standard error values showing the reliability of nodes in** **Figure 5A** **.**
(TXT)

Table S5 **Sequence and position of ELM motifs in representative Myc proteins and the positions in the ClustalW multiple alignment.**
(CSV)

Table S6 **Position of ANCHOR regions in human c-Myc (P01106) and the number of different ELMs they contain.**
(CSV)

Table S7 **Position of ANCHOR regions in human MycN (P04198) and the number of different ELMs they contain.**
(CSV)

Table S8 **Position of ANCHOR regions in human MycL1 (P12524) and the number of different ELMs they contain.**
(CSV)

Table S9 **Position of ANCHOR regions in human MycL2 (P12525) and the number of different ELMs they contain.**
(CSV)
